# Comparison of PBS-Caffeine and Caffeine Buffers for Inhibiting Exocytosis During Horseshoe Crab Blood Collection and Improving the Yield of Limulus Amebocyte Lysate (LAL) for Endotoxin Detection

**DOI:** 10.3390/ijms27125628

**Published:** 2026-06-22

**Authors:** Jessica Zhang, Sophia Zhang, Mengmeng Zhang

**Affiliations:** Department of Biology, Westford Academy, Westford, MA 01886, USA; szhang6781@gmail.com (S.Z.); 29mzhang@gmail.com (M.Z.)

**Keywords:** Limulus amebocyte lysate (LAL), endotoxin detection, horseshoe crab, *Limulus polyphemus*, (1,3)-β-D-glucan, Factor G, caffeine collection buffer, exocytosis

## Abstract

Limulus amebocyte lysate (LAL) detects bacterial endotoxin through a serine-protease coagulation cascade in which Factor C responds to lipopolysaccharide and Factor G to (1,3)-β-D-glucan. Sustainable LAL production depends on collection buffers that prevent amebocyte degranulation while preserving these clotting factors. We previously showed that caffeine buffer inhibits degranulation, but caffeine-collected pellets aggregated upon resuspension in 5 mM CaCl_2_, unlike phosphate-buffered saline (PBS). We therefore developed a PBS-caffeine collection solution and compared it with caffeine buffer. Over one bleeding season, 121 crabs were bled; blood was collected in caffeine, PBS-caffeine, or PBS-caffeine supplemented with EDTA, EGTA, or both, and LAL activity was measured by chromogenic and turbidimetric assays. Both buffers prevented degranulation and gave comparable LAL activity, but PBS-caffeine reduced aggregation and clotting. Treating PBS-caffeine LAL with 10% PEG-8000 selectively abolished endotoxin-sensitive Factor C activity while preserving (1,3)-β-D-glucan–sensitive Factor G activity, and the resulting Factor G lysate, formulated in 20 mM acetate (pH 5.6), remained stable for 27 months. These results define an improved collection buffer and identify conditions that selectively stabilize Factor G zymogen in liquid form.

## 1. Introduction

Quality control is critically important in the pharmaceutical and biotechnology industries. If parenteral pharmaceutical products or implantable medical devices are contaminated with endotoxin and enter the bloodstream, they can cause multi-organ dysfunction and increase the risk of mortality [1].

Endotoxin is a lipopolysaccharide found in the outer membrane of gram-negative bacteria. When introduced into the human body, it can induce fever, shock, or even death. Because of these risks, controlling endotoxin contamination is critical for biopharmaceutical products, particularly those administered intravenously. Limulus amebocyte lysate (LAL)-based assays are commonly used to detect endotoxin in biological formulations [2]. Moreover, because LAL reagents contain Factor G, which is activated by (1,3)-β-D-glucan, a structural component of the fungal cell wall, LAL assays can also aid in the diagnosis of invasive fungal infections [3,4].

The LAL test, which uses horseshoe crab blood, has long been the gold standard for endotoxin detection because of its high sensitivity and specificity [5]. First approved by the FDA in 1977 for endotoxin testing, the LAL method was later adapted for the diagnosis of invasive fungal infection with the introduction of the Fungitell assay in 2004. This assay detects (1,3)-β-D-glucan, a biomarker of invasive fungal infection [6]. Since then, numerous studies have used LAL-based tests to diagnose such infections in a range of clinical samples, including serum [7,8], plasma [9], cerebrospinal fluid (CSF) [10,11], and ocular fluids [12].

LAL is derived from horseshoe crab blood, and its production typically involves three main steps. First, a collection buffer is used to collect the blood, and the mixture is centrifuged to isolate the cell pellets. Next, the pellets are washed, resuspended in a resuspension buffer, and shaken overnight. Finally, LAL is extracted from the supernatant [13].

Procuring blood for the LAL test involves capturing and bleeding more than 500,000 crabs from wild marine populations each year. Although manufacturers attempt to return the crabs to the sea after bleeding, the process still results in some mortality and sublethal effects, raising growing welfare and ethical concerns. The 3Rs principle—which advocates the replacement, reduction, and refinement of animal use in science—is a globally accepted framework for ethical, animal-dependent research [14].

To reduce the impact on crab populations, various methods have been developed for collecting horseshoe crab blood. Armstrong et al. reported using a 3% NaCl solution to collect horseshoe crab blood for studying amebocyte exocytosis [15]. However, our repeated experiments showed that degranulation occurred during collection when 3% NaCl was used as the buffer, resulting in very low enzyme activity in the final LAL. Consequently, this method is unsuitable for large-scale production.

Our previous study demonstrated that an 80 mM caffeine collection buffer (80 mM caffeine, 3% NaCl) prevents degranulation during blood collection and that caffeine-derived LAL functions in both chromogenic and turbidimetric assays [13]. However, cell pellets collected in 80 mM caffeine aggregated upon resuspension in 5 mM CaCl_2_, potentially reducing the recovery of LAL activity. No such aggregation occurred with PBS buffer, suggesting that certain components of the PBS collection buffer inhibit clot formation. We therefore examined the effects of phosphate, EDTA, phosphate plus EDTA, and EDTA plus EGTA on clot formation. Based on these findings, an 80 mM caffeine collection buffer containing buffering and chelating agents was developed and tested, and a PBS-caffeine bleeding solution was ultimately formulated and evaluated [16].

To further compare the inhibitory effects of PBS-caffeine and caffeine buffers on exocytosis during horseshoe crab blood collection, experiments were conducted during the bleeding season. Each week, 6 or 12 crabs were bled, for a total of 121 crabs, using both the PBS-caffeine and caffeine collection solutions. Blood from each crab was collected with the 80 mM caffeine solution, and its derived variants, and the resulting cell pellets were resuspended in 5 mM CaCl_2_ to induce degranulation. Both chromogenic and turbidimetric assays then measured LAL activity.

Notably, all commercial LAL products are supplied as lyophilized powders. However, lyophilization is time-consuming and costly. In addition, users must reconstitute the lyophilized reagent before use, which requires the production and supply of reconstitution fluid, further increasing costs. By contrast, a stable liquid formulation would enable more flexible, cost-effective, high-throughput manufacturing and allow for diverse product presentations.

Our small-scale experiments revealed that a commercial LAL reagent reconstituted in 20 mM acetate (pH 5.6) remained stable for nearly 4 months at 4 °C. A separate stability test showed that citric acid–derived LAL resuspended in 10 mM CaCl_2_ remained stable for one and a half years. Together, these results suggest that an acidic environment combined with Ca^2+^ may help stabilize LAL. Because the key clotting factors are serine-protease zymogens, defining the solution conditions that preserve their native, activatable state is central to producing a stable liquid reagent. To test this, PBS-caffeine LAL resuspended in 5 mM CaCl_2_ was prepared with 10% PEG-8000, stored in 20 mM acetate (pH 5.6), and assayed for activity at various time intervals.

## 2. Results

### 2.1. Comparison of the Effects of PBS and Caffeine Buffers on Degranulation Inhibition During Blood Collection

**Figure 1:**
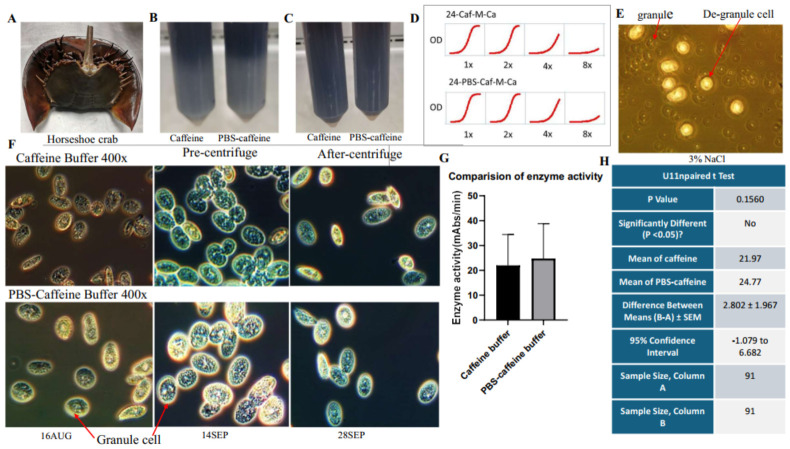
Both the PBS-caffeine and caffeine buffers inhibited exocytosis. (**A**) Horseshoe crab. (**B**) The mixture was incubated at room temperature for 1 h. (**C**) After centrifugation at 1000 rpm at 4 °C for 5 min, (**D**) The enzyme reaction curve at different LAL concentrations was determined using the chromogenic method. (**E**) Degranulation in 3% NaCl solution. (**F**) An amoebocyte observed under a microscope (400× magnification). (**G**,**H**) Comparison of enzymatic activity between PBS-caffeine and caffeine LAL groups (24.77 ± 14.01 vs. 21.97 ± 12.47, *p* = 0.1596).

Previous studies have shown that both caffeine buffers and PBS-caffeine buffers prevent degranulation during blood collection [13,16], with PBS-caffeine buffers appearing slightly more effective and yielding higher LAL enzyme activity. To confirm this, blood was collected from 91 crabs (Figure 1A) using either caffeine buffer or PBS-caffeine buffer.

As shown in Figure 1B,C, both buffers effectively prevented degranulation after the blood–buffer mixture was incubated at room temperature for 1 h, with no clot formation in either mixture. Microscopy confirmed that no granules were released from the amebocytes (Figure 1F), consistent with the visual observations (Figure 1B,C). This consistency was maintained across different collection time points. In contrast, most granules were released when blood was collected with 3% NaCl (Figure 1E).

Enzyme activity was assessed by a chromogenic assay. Both caffeine-LAL and PBS-caffeine-LAL exhibited high activity, requiring at least a fourfold dilution to bring the readings within the analytical range (Figure 1D). Statistical analysis of the fourfold-diluted samples revealed no significant difference between PBS-caffeine-LAL and caffeine-LAL (*p* = 0.156), although the PBS-caffeine group showed slightly higher activity (24.77 vs. 21.97 mAbs/min; Figure 1G,H).

### 2.2. Evaluation of the Effects of PBS-Caffeine Buffers Supplemented with EDTA or EGTA on Exocytosis

Previous studies have shown that both caffeine buffers and PBS buffers inhibit degranulation during blood collection and produce a high yield of LAL [13,16]. To determine which PBS component reduces clot formation during resuspension, individual PBS components were added to the caffeine collection buffer and tested.

**Figure 2:**
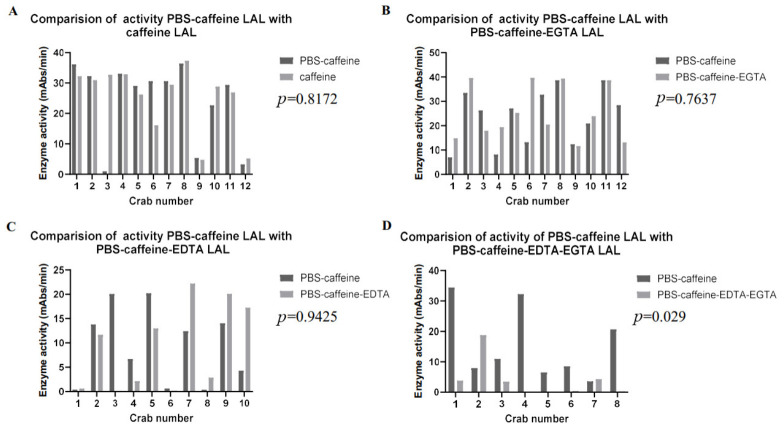
Comparison of enzyme activity across different blood collection buffers. (**A**) PBS-caffeine LAL vs. caffeine LAL (24.13 ± 13.14 vs. 25.27 ± 10.79 mAbs/min, *p* = 0.8172); (**B**) PBS-caffeine LAL vs. PBS-caffeine-EGTA-LAL (23.89 ± 11.41 vs. 25.28 ± 11.06 mAbs/min, *p* = 0.7631); (**C**) PBS-caffeine LAL vs. PBS-caffeine-EDTA-LAL (9.28 ± 7.829 vs. 9.007 ± 8.81 mAbs/min, *p* = 0.9429); (**D**) PBS-caffeine LAL vs. PBS-caffeine-EGTA-EDTA-LAL (15.59 ± 12.04 vs. 3.909 ± 6.268 mAbs/min, *p* = 0.029).

Blood was collected from 12 crabs using either PBS-caffeine buffer (without EDTA or EGTA) or caffeine buffer alone. After a single wash with 3% NaCl, the cell pellets were resuspended in 5 mM CaCl_2_. Clotting was almost eliminated when pellets collected with PBS-caffeine buffer were resuspended in 5 mM CaCl_2_. Enzyme activity was then measured by the chromogenic method. Because of the high total activity, the raw LAL required a fourfold dilution for accurate measurement (Figure 1D); the diluted results, which fell within the optimal analytical range, were used for statistical analysis. Figure 2A shows no significant difference between the PBS-caffeine and caffeine buffers (24.13 vs. 25.27 mAbs/min, *p* = 0.8172), consistent with Figure 1G,H.

To assess whether EGTA supplementation of PBS-caffeine buffer could further reduce clot formation and improve LAL yield, blood from 12 crabs was collected in either PBS-caffeine or PBS-caffeine-EGTA buffer, and the cell pellets were resuspended in 5 mM CaCl_2_ as before. No additional reduction in cell aggregation was observed visually, and enzyme activity (Figure 2B) showed no significant difference between the two buffers (23.89 vs. 25.28 mAbs/min, *p* = 0.7637).

Next, we tested whether EDTA supplementation of PBS-caffeine buffer could enhance clot prevention and increase LAL yield. Blood from 10 crabs was collected in either PBS-caffeine or PBS-caffeine-EDTA buffer. After resuspension in 5 mM CaCl_2_, no further reduction in cell aggregation was observed, and enzyme activity (Figure 2C) showed no clear benefit from EDTA (9.28 vs. 9.007 mAbs/min, *p* = 0.9425).

Finally, we evaluated whether adding both EDTA and EGTA to PBS-caffeine buffer could further improve results. Blood from 8 crabs was collected in either PBS-caffeine or PBS-caffeine-EGTA-EDTA buffer. After resuspension in 5 mM CaCl_2_, no additional reduction in cell aggregation was observed; however, enzyme activity (Figure 2D) differed significantly between the two buffers (15.59 vs. 3.909 mAbs/min, *p* = 0.029).

These results suggest that neither EDTA nor EGTA enhances the ability of PBS-caffeine buffer to prevent degranulation. The underlying mechanism remains unclear and warrants further investigation.

### 2.3. Evaluation of the Stability of Caffeine-LAL and PBS-Caffeine-LAL in Different Resuspension Solutions

To assess the stability of caffeine-LAL and PBS-caffeine-LAL, blood was collected from three crabs of different sizes: one large (15–20 lb), one medium (10–15 lb), and one small (5–10 lb). Blood from each crab was collected into four Corning tubes, each containing 15 mL of buffer, for a total of 60 mL of blood per crab; the same procedure was used for PBS-caffeine buffer.

The blood buffer mixture was processed as described in the Materials and Methods. The resulting cell pellets were resuspended in LRW, 5 mM CaCl_2_, 5 mM MgCl_2_, or 5 mM NaCl and agitated overnight. The supernatant was collected in sterile glass tubes and stored at 4–8 °C. Enzyme activity was measured at various time intervals using the same endotoxin concentration, procedure, and plate reader.

**Figure 3:**
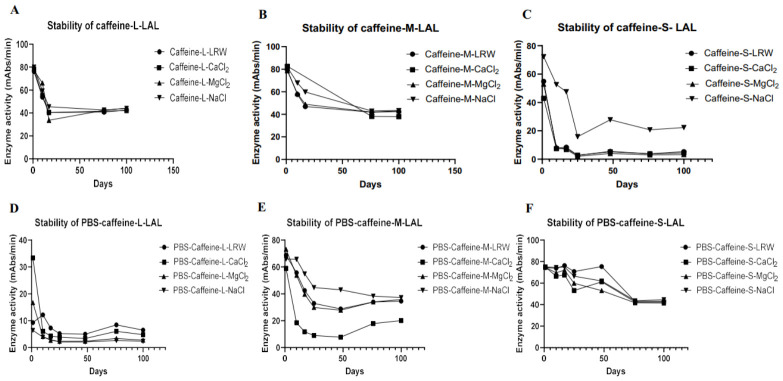
Stability of caffeine-LAL and PBS-caffeine-LAL in different resuspension solutions (LRW, 5 mM CaCl_2_, 5 mM MgCl_2_, and 5 mM NaCl). (**A**) Stability of caffeine buffer-large crab-LAL; (**B**) Stability of caffeine buffer-middle crab-LAL; (**C**) Stability of caffeine buffer-small crab-LAL; (**D**) Stability of PBS-caffeine buffer-large crab-LAL; (**E**) Stability of PBS-caffeine buffer-middle crab-LAL; (**F**) Stability of PBS-caffeine buffer-small crab-LAL.

The results (Figure 3) indicated that neither caffeine-LAL nor PBS-caffeine-LAL remained stable, and the resuspension solution had no significant effect on LAL stability. Approximately 50% of the enzyme activity was lost in caffeine-LAL from the large crab (Figure 3A), caffeine-LAL from the medium-sized crab (Figure 3B), and PBS-caffeine-LAL from the medium-sized crab (Figure 3E). In contrast, more than 80% of the activity was lost in caffeine-LAL from the small crab (Figure 3C) and PBS-caffeine-LAL from the large crab (Figure 3D). LAL was more stable in the PBS-caffeine small-crab preparation (Figure 3F), indicating considerable variability in stability among LAL preparations from different crabs.

### 2.4. Comparison of the Ability of PBS-Caffeine Collection Buffer and Caffeine Collection Buffer to Prevent Degranulation Across Different Bleeding Seasons and Vendors

To evaluate the effects of PBS-caffeine and caffeine collection buffers on degranulation and LAL yield across different months and vendors, 118 crabs were bled from May to September: 24 in May 28 in June 13 in July 29 in August, and 24 in September. Blood was collected with both buffers, and the cell pellets were resuspended in 5 mM CaCl_2_ for degranulation analysis. Crabs from all four vendors were included.

Both buffers effectively prevented degranulation for at least 3–4 h during blood collection. Figure 1F illustrates the non-degranulated state of the amebocytes, which was maintained for this period after collection.

**Figure 4:**
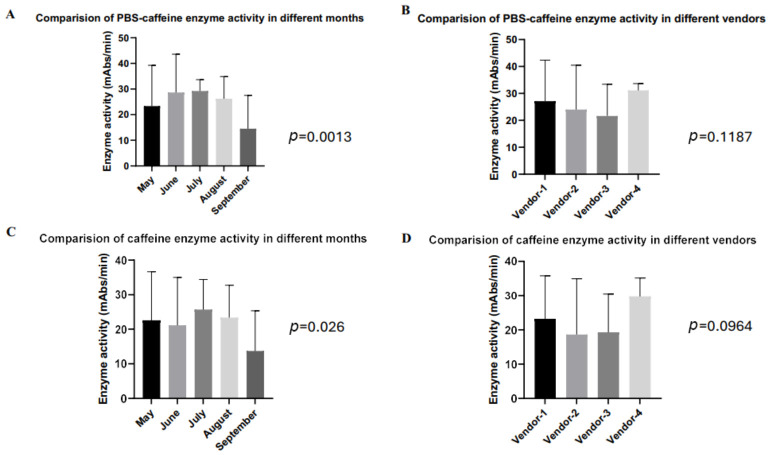
Comparison of PBS-caffeine-LAL and caffeine-LAL in different months and vendors. (**A**) Comparison of the enzyme activity of PBS-caffeine-LAL in different months (The enzyme activities in May, June, July, August, and September were 23.33 ± 15.93, 28.64 ± 14.97, 29.25 ± 4.411, 26.25 ± 8.59, and 14.52 ± 13.13 mAbs/min, respectively, *p* = 0.0013). (**B**) Comparison of the enzyme activity of PBS-caffeine-LAL from different vendors (the enzyme activities from vendor-1, vendor-2, vendor-3, and vendor-4 were 27.15 ± 15.21, 24.03 ± 16.44, 21.65 ± 11.74, and 31.1 ± 2.567 respectively, *p* = 0.1187). (**C**) Comparison of the enzyme activity of caffeine-LAL in different months (the enzyme activities in different months were 22.58 ± 14.06, 21.14 ± 13.88, 25.69 ± 8.701, 23.4 ± 9.352, and 13.71 ± 11.64, respectively, *p* = 0.026). (**D**) Comparison of the enzyme activity of caffeine-LAL from different vendors (the enzyme activities in different vendors were 23.21 ± 12.6, 18.64 ± 16.29, 19.28 ± 11.16, and 29.77 ± 5.372, respectively, *p* = 0.0964).

LAL activity was higher from May to August than in September for both the PBS-caffeine buffer (mean activity 23.33, 30.675, 28.692, 26.373, and 14.613 mAbs/min in May, June, July, August, and September, respectively; *p* = 0.0013) and the caffeine buffer (mean activity 22.581, 22.305, 24.807, 23.432, and 13.492 mAbs/min, respectively; *p* = 0.026). This suggests that crabs collected earlier in the season (May–August) may have been in better physiological condition than those collected in September (Figure 4A,C).

To assess vendor-related effects on LAL activity, blood was collected from crabs supplied by four vendors (vendor 1, 36 crabs; vendor 2, 64 crabs; vendor 3, 16 crabs; vendor 4, 8 crabs) using both buffers. After resuspension in 5 mM CaCl_2_, enzyme activity was measured by the chromogenic method. As shown in Figure 4B,D, activity was slightly higher for vendors 1 and 4 than for vendors 2 and 3 for both PBS-caffeine-LAL (mean activity 29.055, 23.998, 26.57, and 31.095 mAbs/min, respectively; *p* = 0.1187) and caffeine-LAL (mean activity 23.52, 18.968, 20.15, and 29.634 mAbs/min, respectively; *p* = 0.0964). These differences may reflect variation in crab husbandry practices among vendors.

### 2.5. Evaluation of the Effects of Caffeine and CaCl_2_ on Enzyme Activity and the Role of CaCl_2_ in Exocytosis

**Figure 5:**
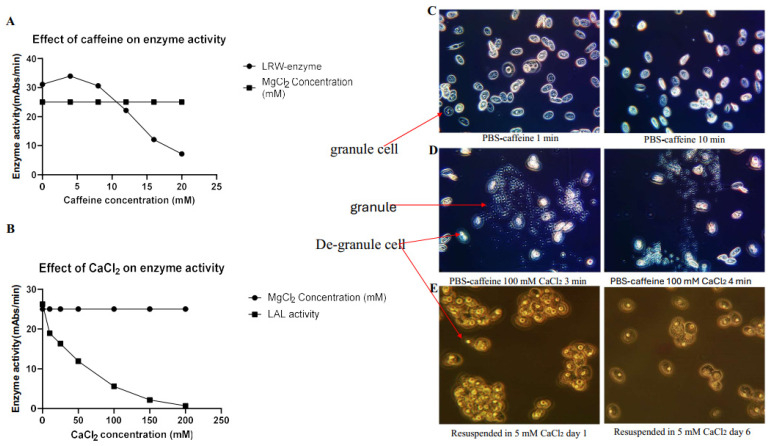
(**A**) Effect of caffeine buffer on enzyme activity. Different final concentrations of caffeine (0, 4, 8, 12, 16, and 20-mM) were incubated with 50 µL of LAL for 5 min before adding the reaction mixture. The plate was then placed in a plate reader, and the reaction was monitored at 37 °C for 1 h. (**B**) Effect of CaCl_2_ on enzyme activity. Various final concentrations of CaCl_2_ (0, 10, 25, 50, 100, 150, and 200 mM) were incubated with 50 µL of LAL for 5 min, followed by the addition of the reaction mixture. The plate was placed in a plate reader, and the reaction was monitored at 37 °C for 1 h. (**C**) Microscopic observation of amoebocyte (400× magnification). After blood collection with PBS-caffeine buffer, the mixture was incubated at room temperature. At 1 min and 10 min, 15 µL of the hemolymph mixture was placed on an endotoxin (LPS)-free glass slide and covered with an LPS-free coverslip. The slide was immediately observed under a microscope to examine granulocytes (10 × 40 magnification). (**D**) Effect of CaCl_2_ on granulocytes. To assess CaCl_2_-induced exocytosis, 100 mM CaCl_2_ was mixed with a PBS-caffeine hemolymph solution on an LPS-free glass slide and covered with an LPS-free coverslip, the degranulation process was monitored via microscopy, and images were captured at different time intervals using a microscope-mounted camera. (**E**) Microscopic observation of amoebocytes after resuspension in 5 mM CaCl_2_. Amoebocytes were examined under a microscope after being resuspended in 5 mM CaCl_2_ for 1 or 6 days.

Our previous study suggested that caffeine buffer may inhibit degranulation, possibly by reducing the free Ca^2+^ available for exocytosis, although this mechanism remains to be confirmed [13]. To investigate this further, we incubated various concentrations of caffeine with LAL in a 96-well plate for 5 min before adding the reaction mixture, and enzyme activity was measured by a chromogenic assay for 1 h. As shown in Figure 5A, enzyme activity decreased progressively with increasing caffeine concentration. Higher concentrations of CaCl_2_ similarly reduced enzyme activity (Figure 5B). MgCl_2_ was held at 25 mM in both conditions (Figure 5A,B).

Interestingly, when the PBS-caffeine–blood mixture was incubated with 100 mM CaCl_2_ for just 3–4 min, nearly complete granule release was observed (Figure 5D). In contrast, no degranulation occurred in samples treated with PBS-caffeine alone, even after 10 min (Figure 5C). Moreover, degranulated cells remained intact after resuspension in 5 mM CaCl_2_ for 6 days (Figure 5E), confirming that the process involves exocytosis rather than cell lysis.

### 2.6. Evaluation of PBS-Caffeine-LAL and Caffeine-LAL Activity at Low Endotoxin Concentrations

**Figure 6:**
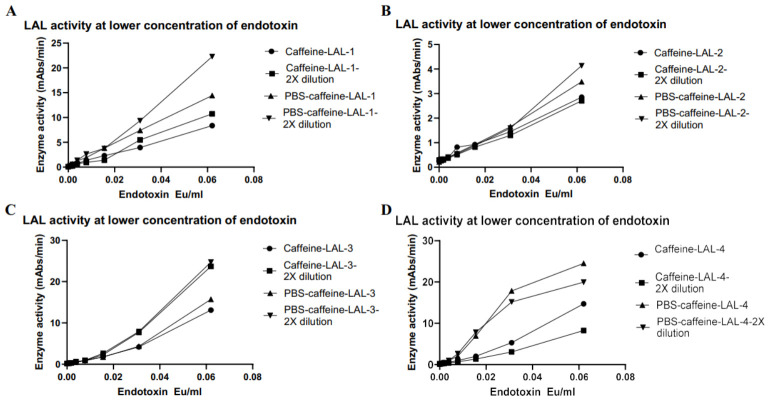
Both PBS-caffeine LAL and caffeine-LAL are functional at low endotoxin concentrations. Blood was collected from four crabs using either PBS-caffeine or caffeine collection buffer. The samples were resuspended in a 5 mM CaCl_2_ solution and shaken overnight at 4 °C. LAL activity was then tested at low endotoxin concentrations (0.0009 to 0.062 Eu/mL). (**A**) Enzyme activity of PBS-caffeine-LAL-1 and caffeine-LAL-1, along with their twofold dilutions, at low endotoxin concentrations. (**B**) Enzyme activity of PBS-caffeine-LAL-2 and caffeine-LAL-2, along with their twofold dilutions, at low endotoxin concentrations. (**C**) Enzyme activity of PBS-caffeine-LAL-3 and caffeine-LAL-3, along with their twofold dilutions at low endotoxin concentrations. (**D**) Enzyme activity of PBS-caffeine-LAL-4 and caffeine-LAL-4, along with their twofold dilutions at low endotoxin concentrations.

To determine whether PBS-caffeine-LAL and caffeine-LAL remain sufficiently active at low endotoxin concentrations (0.001–0.062 EU/mL), blood from four crabs was collected in PBS-caffeine and caffeine collection buffer, respectively. The samples were resuspended in 5 mM CaCl_2_, shaken overnight at 4 °C, and tested for LAL activity at low endotoxin concentrations (Figure 6).

Both PBS-caffeine-LAL and caffeine-LAL showed higher activity at lower endotoxin concentrations. Notably, the activity of both lysates (Figure 6A–C) increased after a twofold dilution, suggesting the possible presence of inhibitors in these preparations.

### 2.7. Evaluation of Factor G Lysate Stability

**Figure 7:**
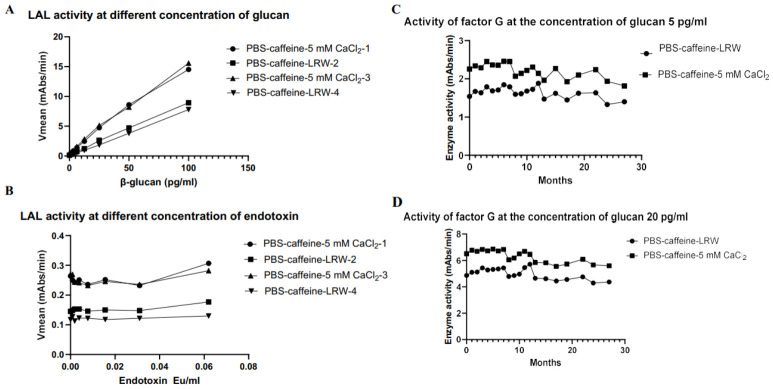
Factor G lysate activity at different concentrations of endotoxin and (1,3)-β-D-glucan, as well as Factor G stability at 4 °C. The Factor G lysate was prepared as described in the Section Materials and Methods. Factor G activity was assessed in the presence of endotoxin or (1,3)-β-D-glucan using a chromogenic assay. (**A**) Lysate activity of four different Factor G lots in response to (1,3)-β-D-glucan. (**B**) Lysate activity of four different Factor G lots in response to endotoxin. (**C**) Factor G lysate activity over time in the presence of 20 pg/mL (1,3)-β-D-glucan. (**D**) Factor G lysate activity over time in the presence of 5 pg/mL (1,3)-β-D-glucan.

To assess the stability of Factor G lysate, the lysate was prepared as described in the Materials and Methods, and its activity was tested with both β-glucan and endotoxin by the chromogenic method in a plate reader for 1 h. The endotoxin-related activity of the prepared Factor G lysate was nearly eliminated (Figure 7B), whereas the (1,3)-β-D-glucan-related activity remained intact (Figure 7A).

To further investigate the stability of the Factor G lysate prepared from PBS-caffeine LAL, the lysate was formulated in 20 mM acetate buffer (pH 5.6) containing 10% PEG-8000. A 400 µL aliquot was dispensed into sterile glass vials and stored at 4 °C. Each month, one vial was tested for (1,3)-β-D-glucan reactivity at final concentrations of 5 and 20 pg/mL, as described in the Materials and Methods. As shown in Figure 7C,D, more than 80% of the enzyme activity was retained after 27 months of storage.

## 3. Discussion

This study demonstrates that both caffeine and PBS-caffeine buffers inhibit amebocyte exocytosis during bleeding and yield highly active LAL suitable for endotoxin detection and β-glucan testing. Although the two buffers gave statistically comparable LAL activity, the PBS-caffeine buffer reduced cell aggregation and clotting during processing, which simplifies handling and recovery. Factor G lysate formulated in 20 mM acetate (pH 5.6) with 10% PEG-8000 remained stable for 27 months, identifying conditions that selectively stabilize the Factor G zymogen in liquid form. Together, these advances could reduce the number of horseshoe crabs required and support more sustainable, cost-effective LAL manufacturing.

The LAL test is the gold standard for endotoxin detection, valued for its sensitivity and specificity, and is endorsed by the U.S. Pharmacopeia and the FDA [1,6]. It is used to release oral and injectable medicines, USP-grade water, and implantable devices [6,17], and is applied beyond healthcare in food, nanomaterial, and occupational-safety testing [18,19,20].

LAL reagents are aqueous extracts of amebocytes from the horseshoe crab (*Limulus polyphemus*). These reagents react with bacterial endotoxin to form a semisolid mass (coagulation) through their intrinsic clotting factors. This reaction is the basis of the LAL test methods widely used in industry. Biochemical studies of the *Limulus* test mechanism have shown that amebocytes contain six protein components: coagulogen, the proclotting enzyme, Factor C, Factor B, and Factor G (which comprises α and β subunits). These proteins form a coagulation cascade triggered by either endotoxin or (1,3)-β-D-glucan. Endotoxin activates Factor C, whereas (1,3)-β-D-glucan activates Factor G. Both pathways converge on the proclotting enzyme, leading to its activation and the subsequent hydrolysis of chromogenic peptide substrates [21].

LAL endotoxin assays are performed in three formats: the gel-clot test, a visual endpoint in which a firm gel withstands inversion of the tube through 180°; the turbidimetric assay, which follows the change in light scattering in real time; and the chromogenic assay, in which the activated clotting enzyme releases a chromophore that is quantified with a plate reader [1].

Because LAL reagents contain Factor G, which responds to (1,3)-β-D-glucan, they also support the diagnosis of invasive fungal infections [3,4,7], including candidiasis [7,22], aspergillosis [23], Pneumocystis jirovecii pneumonia [24], and infections in immunocompromised patients [25], as well as Coccidioides [11] and cryptococcal [10] meningitis—where (1,3)-β-D-glucan may carry prognostic value [10]—and, more recently, fungal endophthalmitis [12].

Beyond classical microbiology, circulating (1→3)-β-D-glucan (BDG) and lipopolysaccharide (LPS) are increasingly studied as markers of gut-barrier compromise and microbial translocation. In critically ill patients, BDG from colonizing fungi can enter the systemic circulation and has been associated with host inflammation and clinical outcomes [9], and BDG is emerging as an important trigger of monocyte and NK-cell activation [26].

Elevated plasma BDG has further been linked to respiratory morbidity in COPD [27] and, in HIV infection, to gut integrity and microbial translocation [28] and to neurocognitive performance [29,30]. These expanding clinical applications underscore the growing demand for high-quality LAL reagents.

LAL is typically produced in three main steps. First, horseshoe crab blood is collected in a collection buffer. The blood–buffer mixture is then centrifuged to isolate the cell pellets, which are washed, resuspended in a resuspension buffer, and shaken overnight at 4 °C. Finally, LAL is extracted from the supernatant of the lysed pellet–buffer mixture. However, procuring blood for the LAL test involves capturing and bleeding more than 500,000 wild horseshoe crabs each year, many of which die in the process. According to the Atlantic States Marine Fisheries Commission (ASMFC)—the body that regulates horseshoe crab populations—the estimated mortality rate applied to bled and released crabs is 15% [14]. Given the rising global demand for pharmaceuticals, the continued and potentially growing use of horseshoe crabs has raised significant concerns about their welfare and long-term sustainability.

The replacement, reduction, and refinement of animal use—collectively known as the “3Rs”—are globally accepted as the framework for governing animal-dependent science, balancing high ethical standards with high-quality research. Because a substantial fraction of granules is lost during conventional bleeding, reducing granule loss at collection and promoting controlled exocytosis during resuspension are the principal levers for improving LAL yield.

Armstrong reported collecting horseshoe crab blood with 3% NaCl [15]. However, our repeated experiments showed that this method caused significant granule loss during collection, making it unreliable for manufacturing. In contrast, citric acid buffer prevents degranulation and yields high LAL activity in the chromogenic assay, although activity is reduced in the turbidimetric assay [16]. Citric, malic, and lactic acids are all carboxylic acids that differ in their number of carboxyl groups: citric acid [HOOC-COH-(CH_2_COOH)_2_] has three, malic acid (HOOC-CHOH-CH_2_COOH) has two, and lactic acid (HOOC-CHOH-CH_3_) has one. Calcium-chelating strength increases with the number of carboxylate groups. Like citric acid, malic and lactic acid buffers yield high LAL activity in the chromogenic assay but lower activity in the turbidimetric assay [16]. By adjusting the pH, we found that buffers with pH > 5.6 retained LAL activity in the turbidimetric assay. For further studies, we selected PBS buffer (pH 5.5–7.0) because of its strong buffering capacity. The formulation contained 20 mM EGTA (Ca^2+^ chelation), 10 mM EDTA (Mg^2+^ chelation), 3% NaCl (osmotic balance), and 100 mM glucose (energy for cell metabolism, including exocytosis). PBS buffer effectively prevented exocytosis and produced a high yield of LAL [16]; importantly, PBS-derived LAL performed well in both the chromogenic and turbidimetric assays.

Our previous study demonstrated that caffeine buffer prevents degranulation and produces a high yield of LAL [13]. However, when cell pellets collected in caffeine buffer were resuspended in 5 mM CaCl_2_, they often clotted and adhered to the walls of the Corning tubes, making it difficult to transfer aliquots into Erlenmeyer flasks and reducing LAL yield. This problem was not observed with PBS buffer. To address this, we tested whether PBS components could reduce clot formation in caffeine-treated samples, leading to the development of the PBS-caffeine buffer [16]. Phosphate addition markedly reduced clotting, and PBS-caffeine buffer produced a marginally higher LAL yield than caffeine buffer, although the difference was not statistically significant (Figure 1G). Adding EGTA, EDTA, or both did not further reduce clot formation (Figure 2B,C), and activity recovery decreased when both were added (Figure 2D). Both PBS-caffeine-LAL and caffeine-LAL performed well in the chromogenic and turbidimetric assays [13,16].

Notably, neither PBS-caffeine-LAL nor caffeine-LAL was stable at 4 °C when resuspended in LRW, 5 mM CaCl_2_, 5 mM MgCl_2_, or 5 mM NaCl (Figure 3). Interestingly, PBS-caffeine-LAL retained Factor G activity, whereas Factor C activity was almost eliminated after treatment with 10% PEG-8000 (Figure 7A,B). The molecular basis of this selective effect remains to be defined. Still, it may reflect differences in the size, domain architecture, and solubility of the two zymogens under PEG-induced molecular crowding: Factor C is a large, multidomain LPS-sensitive protease, whereas Factor G is a non-covalent α/β heterodimer whose protease (β) subunit appears comparatively resistant to inactivation. The selective preservation of Factor G activity offers a practical route to a (1,3)-β-D-glucan–specific reagent for invasive fungal infection testing.

Factor G activation occurs through intermolecular interactions between Factor G molecules bound to (1,3)-β-D-glucan helices. Factor G is a heterodimer of two non-covalently associated subunits, α and β; the β subunit is a serine-protease zymogen, and binding of the α subunit to (1,3)-β-D-glucan is thought to induce a conformational change that activates β [21]. The proclotting enzyme and Factor B share a similar organization, each comprising a disulfide-knotted (clip) domain in the N-terminal light chain and a C-terminal protease domain [21]. Because detection of (1,3)-β-D-glucan requires Factor G to activate the downstream proclotting enzyme, the retained β-glucan reactivity of our stabilized lysate indicates that both Factor G and the proclotting enzyme remain functional.

At present, commercial LAL products are manufactured as lyophilized powders. However, lyophilization incurs significant manufacturing costs, including capital, space, run-time, and energy. A stable liquid formulation of Factor G could improve productivity by reducing the number of unit operations required. Investigating the stability of Factor G lysate in liquid form may therefore offer opportunities to lower costs and develop new product presentations. Our previous study found that a formulation containing 15% sucrose, 20 mM MES (pH 6.0), 5 mM sodium n-octanoate, 5 mM N-acetyl-L-methionine, and 0.5% glutamic acid failed to stabilize Factor G. However, a small-scale experiment showed that Factor G lysate treated with 10% PEG-8000 in 20 mM acetate buffer (pH 5.6) remained stable for 27 months, suggesting that both the acidic environment and 10% PEG-8000 contribute to Factor G stability. At the molecular level, PEG-8000 may act largely as a preferential-exclusion (excluded-volume) crowding agent that favors the compact, folded conformation of the Factor G zymogen and limits its aggregation; however, PEG can also destabilize proteins by interacting with the unfolded state, so its net effect here remains to be determined. The mildly acidic environment (pH 5.6) likely contributes further to stability, although the precise basis is uncertain. Biophysical characterization of the lysate will be needed to confirm these mechanisms.

This study has several limitations. The differences in LAL activity between the PBS-caffeine and caffeine buffers were not statistically significant; the advantage of PBS-caffeine therefore lies in improved processing—reduced aggregation and clotting—rather than in higher yield, and this handling benefit was assessed qualitatively and should be quantified in future work. Several comparisons were based on small numbers of crabs (*n* = 8–12), and sampling was limited to a single bleeding season with possible vendor-related confounding. Neither caffeine- nor PBS-caffeine-derived whole LAL was stable on storage, losing 50–80% of its activity, and the rise in apparent activity after two-fold dilution points to sample-derived inhibitors that warrant further study. Finally, the 27-month stability of Factor G was demonstrated only at a small scale, and the proposed stabilization mechanism remains to be confirmed biophysically and at a manufacturing scale.

In summary, both caffeine and PBS-caffeine buffers inhibit amebocyte exocytosis during bleeding and yield highly active LAL for endotoxin detection and β-glucan testing, with PBS-caffeine offering improved handling. Factor G lysate formulated in 20 mM acetate (pH 5.6) with 10% PEG-8000 remained stable for 27 months. Building on established LAL workflows, these approaches could reduce the number of horseshoe crabs required and support more sustainable, cost-effective reagent production. Recombinant Factor C offers an animal-free alternative for endotoxin detection; the methods described here are complementary, improving the sustainability and economics of crab-derived LAL, where it remains in use.

## 4. Materials and Methods

### 4.1. Amebocyte Collection Methods

The following amebocyte collection solutions were used: PBS buffer (25 mM KH_2_PO_4_, 25 mM Na_2_HPO_4_, 3% NaCl, 100 mM glucose, 20 mM EGTA, 10 mM EDTA, pH 6.0); 80 mM caffeine buffer (3% NaCl, 80 mM caffeine); PBS-caffeine buffer (25 mM KH_2_PO_4_, 25 mM Na_2_HPO_4_, 3% NaCl, 100 mM glucose, 80 mM caffeine, pH 6.0); PBS-caffeine-EGTA buffer (25 mM KH_2_PO_4_, 25 mM Na_2_HPO_4_, 3% NaCl, 100 mM glucose, 20 mM EGTA, 80 mM caffeine, pH 6.0); PBS-caffeine-EDTA buffer (25 mM KH_2_PO_4_, 25 mM Na_2_HPO_4_, 3% NaCl, 100 mM glucose, 10 mM EDTA, 80 mM caffeine, pH 6.0); and PBS-caffeine-EGTA-EDTA buffer (25 mM KH_2_PO_4_, 25 mM Na_2_HPO_4_, 3% NaCl, 100 mM glucose, 20 mM EGTA, 10 mM EDTA, 80 mM caffeine, pH 6.0). The cell-washing solution was 3% NaCl, and the cell-resuspension solutions were LAL reagent water (LRW) and 5 mM CaCl_2_. Blood from American horseshoe crabs (Limulus polyphemus) was provided by a manufacturer of LAL reagent (Boston, MA, USA). No ethics approval was required for this study.

### 4.2. Evaluation of the Effects of Different Blood Collection Solutions on Degranulation Inhibition During Hemolymph Collection

To evaluate the effects of different collection buffers on the inhibition of degranulation during hemolymph collection, 25 mL of hemolymph was collected from each crab and mixed with an equal volume of collection buffer; for comparison, 50 mL of hemolymph was collected from each crab using both solutions. The blood buffer mixture was incubated at room temperature for 1 h and then centrifuged at 1000 rpm (180× *g*) at 10 °C for 5 min. The supernatant was removed, 25 mL of washing buffer was added, and the mixture was centrifuged again under the same conditions; the supernatant was decanted, and the washing step was repeated. The pellet was resuspended in resuspension buffer at a 1:8 ratio (1 g pellet to 8 mL buffer) and vortexed for 30 s. The mixture was transferred to a 50 mL Erlenmeyer flask and shaken at 100 rpm overnight at 4 °C. After shaking, the gel clot settled at the bottom, and the supernatant was transferred to a sterile glass tube for storage. Enzyme activity was assessed by the chromogenic method [13].

Comparison of Different Buffers on Exocytosis Inhibition

1. PBS-caffeine vs. caffeine buffer: hemolymph from 91 crabs was collected using both buffers. Enzyme activity was measured by chromogenic testing, and mean values were compared using GraphPad Prism 10.2.
2. PBS-caffeine vs. PBS-caffeine-EDTA: hemolymph from 10 crabs was tested in the same way.
3. PBS-caffeine vs. PBS-caffeine-EGTA: hemolymph from 12 crabs was analyzed.
4. PBS-caffeine vs. PBS-caffeine-EGTA-EDTA: hemolymph from 8 crabs was evaluated.

In each case, enzyme activity was determined by chromogenic assay, and statistical comparisons were performed using GraphPad Prism 10.2.

### 4.3. Microscopic Examination of Hemocytes

After blood was collected with PBS-caffeine buffer, caffeine buffer, or 3% NaCl, 15 µL of the hemolymph mixture was placed on an endotoxin (LPS)-free glass slide and covered with an LPS-free coverslip. The slide was immediately examined under a microscope (10 × 40 magnification) to observe granulocytes. To assess the effect of CaCl_2_ on exocytosis, 100 mM CaCl_2_ was mixed with the PBS-caffeine hemolymph solution on an LPS-free glass slide, covered with an LPS-free coverslip, and observed under the microscope. The degranulation process was monitored, and images were captured at various time intervals with a camera attached to the microscope.

To examine changes in cell morphology after degranulation, a small portion of the cell pellet was resuspended in 5 mM CaCl_2_ and gently shaken at 4 °C. On days 1 and 6, 20 µL of the suspension was placed on an endotoxin-free glass slide and observed under the microscope to monitor amebocyte morphology.

### 4.4. Chromogenic Methods

Activity analysis was performed in 96-well plates as previously described [13]. LAL was serially diluted twofold (from 1:2 to 1:16) in LAL reagent water (LRW). A 50 µL aliquot of each dilution was added in triplicate to the wells, followed by 50 µL of reaction mixture (0.14 M Tris, pH 7.4, 50 mM MgCl_2_, 6.25 mM chromogenic peptide, and 5 EU/mL endotoxin). The plate was loaded into a BioTek ELx808 plate reader (BioTek Instruments, Winooski, VT, USA) and absorbance was measured at 405 nm at 37 °C for 60 min. The mean reaction rate (Vmean) was analyzed; only values with a coefficient of variation (CV) < 20% were considered valid. This parameter was used to compare enzyme activity across reaction mixtures.

### 4.5. Evaluation of the Effect of the Resuspension Solution on the Stability of the LAL

To determine which resuspension solution best maintained LAL stability, blood was collected in both caffeine buffers and PBS-caffeine buffers. For each crab, blood was drawn into four 50 mL Corning tubes, each containing 15 mL of buffer; approximately 15 mL of blood was added per tube, for a total of 30 mL per tube. The blood buffer mixture was incubated at room temperature for 30 min and then centrifuged at 1000 rpm (180× *g*) at 10 °C for 5 min. After the supernatant was removed, 25 mL of washing buffer was added, and the mixture was centrifuged again under the same conditions; this washing step was repeated once. The cell pellets were resuspended in LRW, 5.0 mM CaCl_2_, 5.0 mM MgCl_2_, or 5.0 mM NaCl at a 1:8 ratio (1 g pellet per 8 mL buffer). The suspensions were vortexed for 30 s, transferred to a 50 mL glass flask, and shaken at 4 °C overnight. The supernatant was collected in a sterile glass tube and stored at 4 °C, and enzyme activity was assessed at various time intervals by the chromogenic method.

### 4.6. Evaluation of the Effects of Caffeine and CaCl_2_ on Enzyme Activity

To assess the effect of caffeine on enzyme activity, LAL (50 µL) was incubated with 0, 4, 8, 12, 16, or 20 mM caffeine in a 96-well plate for 5 min. The reaction mixture (140 mM Tris-Cl, pH 7.4, 50 mM MgCl_2_, 6.25 mM substrate, and 5 EU/mL endotoxin) was then added, and the chromogenic reaction was monitored for 1 h at 37 °C.

To further examine the effect of CaCl_2_ on enzyme activity, LAL (50 µL) was incubated with 0, 5, 25, 50, 100, 150, or 200 mM CaCl_2_ for 5 min before the same reaction mixture was added. The chromogenic reaction was monitored for 1 h at 37 °C.

### 4.7. Factor G Lysate Preparation

A 10% PEG-8000 solution was prepared in sterile water and filtered through a 25 mm acrylic syringe filter with a 0.2 µm Posidyne membrane to remove LAL-activating contaminants. PBS-caffeine LAL was then mixed with the 10% PEG-8000 solution at a 1:1 (*v*/*v*) ratio in 10 mM MES (pH 6.0). The mixture was shaken at room temperature for 15 min and filtered five times through a 5 mL centrifuge tube fitted with a 0.2 µm nylon membrane (Sigma, St. Louis, MO, USA). The glucan-sensitive activity of the filtrate was assessed by the chromogenic method using (1,3)-β-D-glucan (1.56 to 100 pg/mL) and endotoxin (0.0009 to 0.062 EU/mL).

### 4.8. Evaluation of the Stability of the PBS-Caffeine-LRW-LAL-Factor G Lysate and the PBS-Caffeine-5-mM CaCl_2_-LAL-Factor G Lysate

To assess the stability of Factor G lysate, 22.5 mL of either PBS-caffeine-LRW Factor G lysate or PBS-caffeine-5 mM CaCl_2_ Factor G lysate was mixed with 2.5 mL of 200 mM acetate buffer (pH 5.6). A 400 µL aliquot was dispensed into endotoxin-free sterile glass vials. Each month, one vial was tested at a final β-glucan concentration of 5 or 20 pg/mL. Chromogenic assays were performed in triplicate: 50 µL of Factor G lysate was combined with 50 µL of reaction mixture (0.25 M Tris-HCl, pH 7.4, 70 mM MgCl_2_, 0.7 mM chromogenic peptide) in a 96-well plate and incubated for 5 min; 25 µL of β-glucan (100 or 25 pg/mL) was then added, and the reaction was monitored for 1 h. Factor G activity was compared across time intervals.

### 4.9. Statistical Analysis

Two tailed *t*-tests were performed in GraphPad Prism 10.2 to compare the activity of PBS-caffeine-derived LAL with that of caffeine-derived LAL, with significance set at *p* < 0.05. All other statistical analyses were also performed in GraphPad Prism 10.2.

## Data Availability

All data generated or analyzed during this study are included in this published article.
